# *EBF1* gene polymorphism and its interaction with smoking and drinking on the risk of coronary artery disease for Chinese patients

**DOI:** 10.1042/BSR20180324

**Published:** 2018-06-21

**Authors:** Yongjun Ying, Yuxuan Luo, Hui Peng

**Affiliations:** 1 Department of Cardiology, Tongde Hospital of Zhejiang Province, 234 Gucui Road, Hangzhou, Zhejiang, China; 2 Department of Nephrology, Zhuji People’s Hospital of Zhejiang province, Zhuji, Shaoxing, Zhejiang, China

**Keywords:** coronary artery disease, Early B cell factor 1, single nucleotide polymorphism, Sanger sequencing

## Abstract

**Objective**: Early B-cell factor 1 (EBF1) is a transcription factor that is expressed in early B-cells, adipocytes, and olfactory neurons, and is essential for the maturation of early B lymphocytes. The present study analyzes the influence of EBF1 gene polymorphism and its interaction with smoking and drinking on the risk of coronary artery disease (CAD). **Methods:** In the present study, 243 CAD cases were enrolled as the CAD group and 215 non-CAD patients as the control group by case–control study. We analyzed their genotypes of the rs987401919, rs36071027, and rs1056065671 loci of the EBF1 gene by Sanger sequencing and detected their content of HDL-C, LDL-C, and TG. **Results:** The C allele at the rs987401919 and rs36071027 loci of *EBF1* was found to be the risk factor for CAD (Odds ratio, OR = 1.233; 95% confidence interval, CI: 1.039–1.421; *P*=0.017; OR = 1.487; 95% CI: 1.015–1.823; *P*=0.042). The interaction between single nucleotide polymorphisms (SNP) of the rs987401919 and rs36071027 loci and smoking and drinking were distinctly associated with the incidence of CAD (*P*<0.05). The content of systolic blood pressure (SBP), diastolic blood pressure (DBP), HDL-C, LDL-C, and TG was distinctly changed after gene mutation at the rs987401919 and rs36071027 loci (*P*<0.05). **Conclusion**: The results of the present study show that the mutation (CT+TT) at the rs987401919 and rs36071027 loci of *EBF1* and its interaction with smoking and drinking are risk factors for CAD, and that the mechanism may be related to the changes in blood pressure and blood lipid content.

## Introduction

Coronary artery disease (CAD) is a common cardiovascular disease, and its risk factors mainly include immune diseases, chronic inflammation, genetic polymorphisms, environmental factors etc. The interaction between these factors often lead to the formation of coronary atherosclerosis and CAD [1–3]. A series of genetic susceptibility genes related to the risk of CAD were discovered through a Genome-Wide Association Study (GWAS), which will provide valuable clues for clarifying the mechanism of interaction in the pathophysiological process of CAD and between genes and the environment [4,5].

Early B-cell factor 1 (EBF1) is located on human chromosome 5q34, is expressed primarily in early B cells, adipocytes and olfactory neurons, and is a transcription factor essential for B-lymphocyte maturation [6,7]. Recent studies have shown that *EBF1* can regulate inflammation and insulin signaling pathways in adipocytes [8]. *In vivo* experimental studies have shown hypoglycemia, and low-fat metabolic syndrome occurred after knockout of *EBF1* in mice [9]. This evidence suggests that *EBF1* may be associated with atherosclerosis, and there is evidence that EBF1 is a risk factor for CAD [10]. At present, there are few studies on the effect of EBF1 gene polymorphism and its interaction with smoking and drinking on the incidence of CAD. Based on the role of lipid metabolism and coronary atherosclerosis, the rs987401919, rs36071027, and rs1056065671 loci single nucleotide polymorphisms (SNPs) of EBF1 gene were selected for the present study.

## Information and methods

### Clinical information

A total of 243 CAD patients treated in our hospital from October 2014–2017 were enrolled as the CAD group. A total of 215 non-CAD patients were enrolled as the control group, and none of the subjects in this group had more than 20% major coronary stenosis. All CAD patients were confirmed by coronary angiography. Patients with myocardial infarction or coronary angiography showing more than 70% narrowing of at least one of the main branches of the coronary artery were included, while patients younger than 18 years of age and patients with congenital heart disease, cardiomyopathy, valvular heart disease, malignant tumor, or chronic liver and kidney disease were excluded. All the subjects signed the informed consent, and the study was approved by the Medical Ethics Committee of our hospital.

### Determination of biochemical indexes

We collected 10 ml of elbow vein blood (fasting) from each subject. A total of 2 ml was used to extract genomic DNA using the QIAamp DSP DNA Blood Mini Kit (61104, Qiagen, Germany), and was stored at –80°C to be tested. Another 8 ml of whole blood was used to separate the serum (4°C, 3000 rpm, 20 min). The content of HDL-C, LDL-C, and TG were detected using the Hitachi 7076 automatic biochemical analyzer. The HDL-C kit is manufactured by Ek-Bioscience (cat#: EK-H12286), the LDL-C kit by Abcam (cat#: ab14519), and the TG test kit by Abcam (cat#: ab77591). Three readings of both systolic blood pressure (SBP) and diastolic blood pressure (DBP) were averaged for each subject with reference to the standard protocol recommended by the American Heart Association. Hypertension is defined as SBP>140 mm Hg and/or DBP>90 mm Hg or the patient is taking antihypertensive drugs. Smoking is defined as having smoked 100 or more cigarettes in a lifetime. Drinking alcohol is defined as drinking more than 12 times in the previous year.

### Genotyping of SNPs

**Figure 1:**
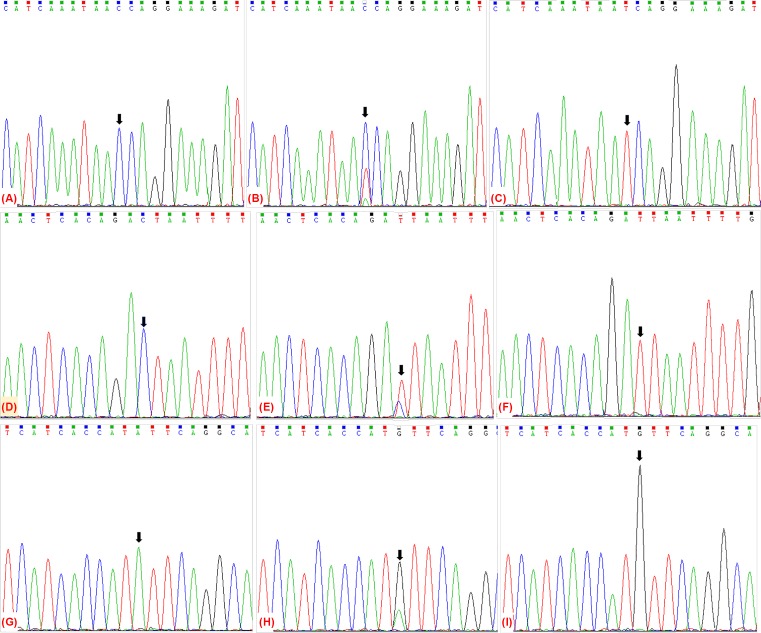
The results of PCR-Sanger sequencing of the *EBF1* gene SNPs (**A**) rs987401919 CC genotype; (**B**) rs987401919 CT genotype; (**C**) rs987401919 TT genotype; (**D**) rs36071027 CC genotype; (**E**) rs36071027 CT genotype;(**F**) rs36071027 TT genotype; (**G**) rs1056065671 AA genotype; (**H**) rs1056065671 GA genotype; (**I**) rs1056065671 GG genotype.

The extracted genomic DNA was amplified by PCR, and the PCR amplification primers for the rs987401919 locus were Forward primer (5′–3′): GCAATTGATCATAAGATAAGAGGCT; Reverse primer (5′–3′: GGCTTAAGAGCAACTTATCACGAA. The PCR amplification primers for the rs36071027 locus were Forward primer (5′–3′): TCTGTGCTGGCTACTTCTCC; Reverse primer (5′–3′): GCCAGTGTGCCTTCTAATGG. The PCR amplification primers for the rs1056065671 locus were Forward primer (5′–3′): TAACAAACAGAGAAGAGGCTAAAAG; Reverse primer (5′–3′): TGCTCTCAGAAGAGGAGATAAAGG. The PCR reaction system includes the following: 1 × PCR buffer, dNTP mix 200 μmol/l, Forward primer and Reverse primer are 10 pmol/l, template gDNA 10 ng, Taq DNA polymerase 2.5 μl, mg^2 +^ 1.5 mmol/l, add sterile water to 25 μl. After amplification, the PCR products were purified by agarose gel electrophoresis, and the sequence of PCR amplification products was detected by Sanger sequencing (see Figure 1).

### Statistical analysis

In the present study, SPSS 20 (SPSS Inc., Chicago, IL, U.S.A.) was used for statistical analysis. The continuous variable is expressed as (± s) and the statistical analysis is student’s *t*test. The counting data were expressed as [*n* (%)] and the statistical analysis was by *χ*^2^ test. Genotype frequencies in both groups were tested by the *χ*^2^ test for Hardy–Weinberg equilibrium. The odds ratio (OR) and 95% confidence interval (CI) were used to analyze the association between genotypes and their interaction with smoking, drinking, and CAD. Factors such as age, sex, BMI, smoking, hypertension, and other factors were corrected by multivariate logistic regression analysis; *P*<0.05 (two-tailed) means the difference was statistically significant.

## Results

### Clinical baseline data

The clinical baseline data of the CAD group and the control group are shown in Table 1. There were no significant differences in age, sex, BMI, SBP, and DBP between the two groups (*P*>0.05). The smoking, drinking, hypertension, LDL-C, and TG of patients in the CAD group were distinctly higher than those in the control group and the level of HDL-C were markedly lower than those in the control group (*P*<0.05). In the present study, there were 77 cases (31.69%) with single-vessel lesions, 82 cases (33.74%) with double-vessel lesions, and 84 cases (34.57%) with triple-vessel lesions in CAD patients.

**Table 1:** Baseline data for the study and control groups

Variable	CAD group (*n=*243)	Control group (*n=*215)	*P* value
Age (years, $\bar{\text{x}}\pm \text{s}$)	63.57 ± 9.90	62.14 ± 9.33	0.114
Male [*n* (%)]	135 (55.56%)	103 (47.91%)	0.102
BMI (kg/m^2^)	24.45 ± 2.66	24.73 ± 2.36	0.237
Smoking [*n* (%)]	89 (36.63%)	52 (24.19%)	0.004
Drinking [*n* (%)]	100 (41.15%)	54 (25.12%)	0.000
Hypertension [*n* (%)]	136 (55.97%)	98 (45.58%)	0.027
Number of vessels diseased [*n* (%)]			
Single vessel	77 (31.69%)		
Double vessels	82 (33.74%)		
Triple vessels	84 (34.57%)		
SBP (mmHg)	128.75 ± 17.62	126.61 ± 25.40	0.302
DBP (mmHg)	76.58 ± 11.65	75.21 ± 12.16	0.219
HDL-C (mmol/l)	1.04 ± 0.28	1.27 ± 0.32	0.000
LDL-C (mmol/l)	2.88 ± 0.43	2.73 ± 0.67	0.005
TG (mmol/l)	1.93 ± 0.79	1.72 ± 0.81	0.005

### The gene frequency of the *EBF1* gene SNP loci and its relationship with the risk of CAD

The distribution of the genotype of the rs987401919, rs36071027, and rs1056065671 loci of *EBF1* in the CAD group and the control group is shown in Table 2. In the present study, the distribution of rs987401919, rs36071027, and rs1056065671 loci in the CAD group and the control group conformed to the Hardy–Weinberg balance (*P*>0.05). The frequency of mutant genotype (CT+TT) of the rs987401919 loci in the CAD group was distinctly higher than that of the control group (27.16–17.21%, *P*=0.011). The gene frequency of mutant genotype (CT+TT) of the rs36071027 loci in the CAD group was distinctly higher than that of the control group (30.04–20%, *P*=0.014). There was no significant difference in the frequency of (GA+AA) at the rs1056065671 locus between the CAD group, and the control group (25.10–23.26%, *P*>0.05). The risk of CAD was distinctly increased after mutation of the C allele to the T allele at the rs987401919 locus (OR = 1.233; 95% CI: 1.039–1.421; *P*=0.017). The risk of CAD was distinctly increased after the rs36071027 C allele mutated to the T allele (OR = 1.487; 95% CI: 1.015–1.823; *P*=0.042). The risk of CAD did not change distinctly after the G allele mutated to the A allele at the rs1056065671 locus (OR = 1.078; 95% CI: 0.895–1.264; *P*=0.440).

**Table 2:** Gene frequencies of the *EBF1* gene, SNPs sites, and their relationship with the risk of CAD

SNPs	CAD group (*n*=243)	Control group (*n=*215)	OR (95% CI)	*P* value	Adjusted OR (95% CI)	*P* value
rs987401919						
Genotype						
CC	177 (72.84%)	178 (82.79%)	Ref			
CT	54 (22.22%)	29 (13.49%)	1.873(1.108–3.174)	0.013	1.305(1049–1.558)	0.018
TT	12 (4.94%)	8 (3.72%)	1.508(0.558–4.151)	0.377	1.203(0.721–1.637)	0.514
Alleles						
C	408 (83.95%)	385 (89.53%)	Ref			
T	78 (16.05%)	45 (10.47%)	1.636(1.085–2.468)	0.013	1.233(1.039–1.421)	0.017
rs36071027						
Genotype						
CC	170 (69.96%)	172 (80.00%)	Ref			
CT	56 (23.05%)	37 (17.21%)	1.531(0.937–2.507)	0.072	1.211(0.99–1.464)	0.093
TT	17 (7.00%)	6 (2.79%)				
Alleles						
C	396 (81.48%)	381 (88.60%)	Ref			
T	90 (18.52%)	49 (11.40%)	2.867(1.032–8.353)	0.025	1.487(1.015–1.823)	0.042
rs1056065671						
Genotype						
GG	182 (74.90%)	165 (76.74%)	Ref			
GA	49 (20.16%)	44 (20.47%)	1.010(0.623–1.638)	0.967	1005(0.786–1.242)	0.999
AA	12 (4.94%)	6 (2.79%)	1.813(0.615–5.561)	0.239	1.271(0.775–1.661)	0.349
Alleles						
G	413 (84.98%)	374 (86.98%)	Ref			
A	73 (15.02%)	56 (13.02%)	1.180(0.798–1.748)	0.386	1.078(0.895–1.264)	0.440

### The influence of the interaction between EBF1 gene and smoking on the risk of CAD

The effect of the interaction between genotypes of EBF1 SNP genotypes and smoking on the risk of CAD is shown in Table 3. The risk of CAD was distinctly higher in smoking subjects with the CT+TT genotype at the rs987401919 locus (OR = 1.521; 95% CI: 1.158–1.847; *P*=0.003), while the risk of CAD in nonsmokers with the CT+TT genotype was not increased (OR = 1.249; 95% CI: 0.920–1.595; *P=*0.156).

**Table 3:** Interaction of smoking, and the genotype of the *EBF1* gene rs987401919, rs36071027, and rs1056065671 loci in the CAD group and the control group

SNPs	Smoking	CAD group (*n* = 243)	Control group (*n* = 215)	OR (95% CI)	*P* value	Adjusted OR (95% CI)	*P* value
rs987401919							
CC	N	122 (50.21%)	140 (65.12%)	Ref			
	Y	55 (22.63%)	38 (17.67%)	1.661(1.001–2.761)	0.037	1.270(1.000–1.564)	0.050
CT+TT	N	32 (13.17%)	23 (10.70%)	1.597(0.853–2.997)	0.117	1.249(0.920–1.595)	0.156
	Y	34 (13.99%)	14 (6.51%)	2.787(1.67–5.746)	0.002	1.521(1.158–1.847)	0.003
rs36071027							
CC	N	112 (46.09%)	130 (60.47%)	Ref			
	Y	58 (23.87%)	42 (19.53%)	1.603(0.975–2.639)	0.049	1.253(0.987–1.551)	0.064
CT+TT	N	42 (17.28%)	33 (15.35%)	1.477(0.850–2.572)	0.141	1.210(0.918–1.533)	0.181
	Y	31 (12.76%)	10 (4.65%)	3.598(1.605–8.243)	0.001	1.634(1.239–1.956)	0.001
rs1056065671							
GG	N	150 (61.73%)	151 (70.23%)	Ref			
	Y	32 (13.17%)	14 (6.51%)	2.301(1.130–4.739)	0.013	1.396(1.058–1.695)	0.019
GA+AA	N	4 (1.65%)	12 (5.58%)	0.336(0.089–1.154)	0.053	0.502(0.166–1.074)	0.093
	Y	57 (23.46%)	38 (17.67%)	1.510(0.921–2.479)	0.084	1.204(0.961–1.462)	0.107

Abbreviations: Y, yes, N, no.

The risk of CAD increased in smoking subjects with the CT+TT genotype at the rs36071027 locus (OR = 1.634; 95% CI: 1.239–1.956; *P*=0.001), whereas the risk of CAD in nonsmoking subjects with the CC genotype and the CT+TT genotype did not distinctly increase (OR = 1.253; 95% CI: 0.987–1.551; *P*=0.064; OR = 1.210; 95% CI: 0.918–1.533; *P*=0.181).

Smoking subjects with the rs1056065671 GG genotype had a higher risk of CAD (OR = 1.396; 95% CI: 1.058–1.695; *P*=0.019), but the risk of CAD in nonsmoking GA+AA genotypes and smoking subjects with the GA+AA genotype was not increased (OR = 0.502; 95% CI: 0.166–1.074; *P*=0.093; OR = 1.204; 95% CI: 0.961–1.462; *P*=0.107).

### The impact of the interaction between *EBF1* and alcohol on the risk of CAD

The effect of the interaction between genotypes of *EBF1*, SNPs, and alcohol consumption on the risk of CAD is shown in Table 4. The risk of CAD in subjects with the rs987401919 locus CT+TT genotype was distinctly higher (OR = 1.477; 95% CI: 1.187–1.760; *P*=0.001). However, the risk of CAD was not distinctly increased in alcohol drinkers with the CC genotype or in nondrinkers with the CT+TT genotype (OR = 1.273; 95% CI: 0.985–1.575; *P*=0.065; OR = 0.972; 95% CI: 0.546–1.457; *P* =0.999). The risk of CAD in drinking subjects with the CT+TT genotype at the rs36071027 locus was distinctly increased (OR = 1.504; 95% CI: 1.215–1.784; *P*=0.000). However, there was no significant change in the risk of CAD among CC-genotype alcohol drinkers and CT+TT–genotype nondrinkers (OR = 1.226; 95% CI: 0.940–1.532; *P*=0.133; OR = 0.938; 95% CI: 0.586–1.346; *P*=0.877). The risk of CAD was distinctly higher in drinking subjects with the rs1056065671 GG genotype and GA+AA genotype (OR = 1.356; 95% CI: 1.094–1.640; *P*=0.005; OR = 1.409; 95% CI: 1.053–1.749; *P* =0.022), but the risk of CAD in nondrinkers with the GA+AA genotype was not distinctly elevated (OR = 0.973; 95% CI: 0.690–1.304; *P* =0.970).

**Table 4:** Interaction of drinking with the *EBF1* rs987401919, rs36071027, and rs1056065671 loci in the CAD group and the control group

SNPs	Drinking	CAD group (*n=*243)	Control group (*n=*215)	OR (95% CI)	*P* value	Adjusted OR (95% CI)	*P* value
rs987401919							
CC	N	132	148	Ref			
	Y	45	30	1.682 (0.971–2.919)	0.048	1.273 (0.985–1.575)	0.065
CT+TT	N	11	13	0.949 (0.381–2.351)	0.902	0.972 (0.546–1.457)	0.999
	Y	55	24	2.569 (1.460–4.542)	0.000	1.477 (1.187–1.760)	0.001
rs36071027							
CC	N	127	141	Ref			
	Y	43	31	1.540 (0.887–2.679)	0.103	1.226 (0.940–1.532)	0.133
CT+TT	N	16	20	0.888 (0.417–1.885)	0.740	0.938 (0.586–1.346)	0.877
	Y	57	23	2.751 (1.553–4.901)	0.000	1.504 (1.215–1.784)	0.000
rs1056065671							
GG	N	114	127	Ref			
	Y	68	38	1.994 (1.213–3.283)	0.040	1.356 (1.094–1.640)	0.005
GA+AA	N	29	34	0.950 (0.525–1.718)	0.857	0.973 (0.690–1.304)	0.970
	Y	32	16	2.228 (1.112–4.502)	0.014	1.409 (1.053–1.749)	0.022

Abbreviations: Y, yes; N, no.

### The relationship between *EBF1* gene polymorphism, and blood pressure and blood lipid content

**Figure 2:**
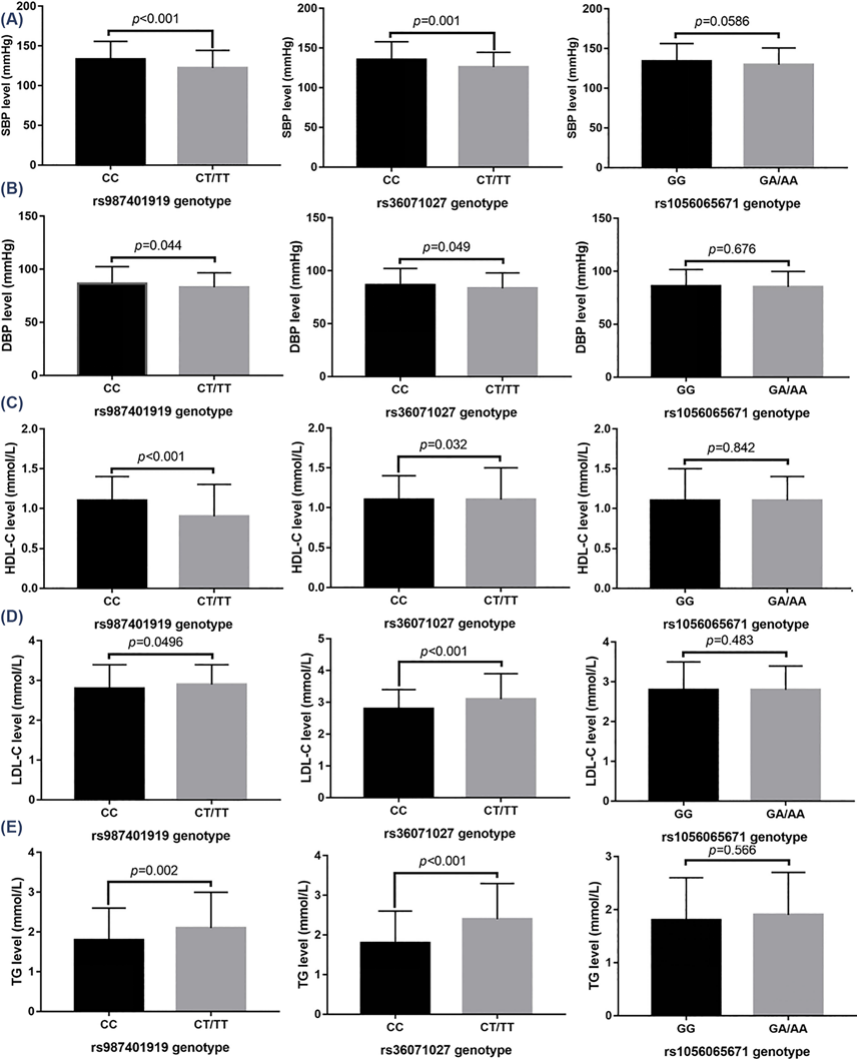
The correlation between the *EBF1* SNPs and the content of SBP, DBP, HDL-C, LDL-C, and TG (**A**) comparison of SBP at each *EBF1* gene SNP site; (**B**) comparison of DBP at each *EBF1* gene SNP site; (**C**) comparison of HDL-C content of *EBF1* SNPs; (**D**) comparison of LDL-C of content *EBF1* SNP; and (**E**) comparison of TG content of *EBF1* SNP.

The relationship between each SNP’s loci genotype of the *EBF1* gene and blood pressure and blood lipid content is shown in Figure 2. The LDL-C and TG content of subjects with rs987401919 mutation (CT+TT) were distinctly higher than those of wild-type (CC) subjects. SBP, DBP, and HDL-C content were distinctly lower than those of wild-type subjects *(P*<0.05). The LDL-C and TG content of subjects with the rs36071027 mutation (CT+TT) were distinctly higher than those of wild-type (CC) subjects. SBP, DBP, and HDL-C content were distinctly lower than those of wild-type subjects (*P*<0.05). There was no difference in the level of SBP, DBP, HDL-C, LDL-C, and TG at the rs1056065671 locus between mutation (GA+AA) subjects and wild-type (GG) subjects (*P* >0.05).

## Discussion

EBF1 is a single-stranded nucleoprotein consisting of 591 amino acids, and is a member of the EBF family of nuclear transcription factors [11]. Studies have shown that *EBF1* plays an important role in the development, differentiation, and expression of the human olfactory nerve and is required for the development of B lymphocytes [12]. The expression of EBF1 mRNA and protein can be detected in all stages of human osteoblast differentiation and in human bone marrow stromal cells. In a study of a mouse model, the number of osteoblasts in the *EBF1* knockout mice increased distinctly [13]. In addition to the development and differentiation of B lymphocytes, the *EBF1* plays an important role in the differentiation of the adipocyte lines [14]. Similarly, after knocking out the *EBF1* in mice, the mice were found to have symptoms such as abnormal fat metabolism, hypoglycemia, and other symptoms [9].

The GWAS study of CAD found that 9p21 in the Iceland and Caucasus groups is susceptible to CAD [15]. GWAS research in China also found that 9p21 and 6p24 had significant correlation with CAD occurrence [16]. Some researchers have confirmed that gene mutation at the rs36071027 locus in *EBF1* resulted in an increase of carotid intima–media thickness, and that carotid intima–media thickness is correlated with CAD severity [17]. Li et al. [18] showed that the risk of CAD in the rs36071027 locus mutant population of the *EBF1* was distinctly increased. The rs987401919, rs36071027, and rs1056065671 loci of the *EBF1* were included in the study and the interaction between the three SNP loci genetic polymorphisms and smoking and alcohol consumption on the risk of CAD was analyzed. The rs987401919 and rs1056065671 loci are located in the downstream regulatory region. Mutations in these loci may affect the expression of *EBF1*, while the rs36071027 locus has been shown to be a risk factor for CAD [18].

The results of present study showed that the frequency of the mutant gene (CT+TT) at the rs987401919 locus in the CAD patient group was distinctly higher than in the control group, and the gene frequency of the rs36071027 locus mutant genotype (CT+TT) was distinctly higher than in the control group. There was no significant difference in the frequency of the rs1056065671 locus between the CAD group and the control group (GA+AA). Further analysis showed that the risk of CAD increased distinctly after rs987401919 and rs36071027 mutation, whereas there was no significant change in the CAD risk after the mutation of G to A at rs1056065671. This shows that the rs987401919 and rs36071027 SNPs distinctly correlate with CAD, which is consistent with the results of the study by Li et al. [18].

Smoking and drinking are risk factors for CAD and have been confirmed in many studies [19,20], but the interaction of *EBF1* with smoking and drinking on the risk of CAD is still rarely reported. The results of present study show that the risk of CAD in smokers with rs987401919 and rs36071027 mutations (CT+TT) was distinctly increased. At the same time, although the rs1056065671 locus GG genotype was not a risk factor for CAD, the risk of CAD was distinctly increased in this genotype in smoking subjects. Similarly, the risk of CAD was distinctly increased in drinkers with the (CT+TT) mutation at the rs987401919 and rs36071027 loci. However, the risk of CAD increased distinctly in subjects with the GG genotype and the GA+AA genotype at the rs1056065671 locus, and the risk of CAD in GA+AA subjects did not increase distinctly, indicating that the interaction between rs1056065671 and alcohol has no significant impact on the risk of CAD.

We also discussed the relationship between EBF1 gene polymorphism and blood pressure and blood lipids. The results showed that the content of LDL-C and TG in the rs987401919 and rs36071027 loci mutant (CT+TT) subjects increased distinctly, whereas SBP, DBP, and HDL-C decreased, indicating that mutations at the rs987401919 and rs36071027 loci may affect the expression of *EBF1*, and thus affect the blood lipid content. Because the rs987401919 site is located at the 3′UTR and rs36071027 is located in the intron region, the authors speculate that these two SNPs sites alter EBF1 protein expression rather than structure and function.

The present study included only Chinese Han population, which excludes the genetic diversity of different races. The results of present study provide some evidence to support the impact of the interaction between EBF1 gene polymorphism and environmental factors on the risk of CAD. However, due to the sample size limitation, the mutant homozygotes are rare in this study. Therefore, the present study combined the mutant heterozygotes with the wild-types to reduce the effect of a too-small sample size on the objectivity of the analysis results.

## Conclusion

The results of our study show that the (CT+TT) mutation at the rs987401919 and rs36071027 loci of EBF1 and its interaction with smoking and drinking are risk factors for CAD. The mechanism may be that the EBF1 gene polymorphisms affect the expression of EBF1 protein, thereby affecting the body’s blood pressure and blood lipid content, but the specific mechanism still needs further study.

## Abbreviations

BMI: body mass index
CAD: coronary artery disease
CI: confidence interval
DBP: diastolic blood pressure
EBF1: early B-cell factor 1
GWAS: Genome-Wide Association Study
HDL-C: high density lipoprotein cholesterol
LDL-C: low density lipoprotein cholesterol
OR: odds ratio
SBP: systolic blood pressure
SNP: single nucleotide polymorphism
TG: triglyceride
